# Cardiac magnetic resonance imaging-derived atrial fibrosis in patients with pre-atrial fibrillation

**DOI:** 10.1136/openhrt-2025-003747

**Published:** 2025-11-27

**Authors:** Ali Wahab, Ramesh Nadarajah, Raluca Tomoaia, Wasim Javed, Catherine Reynolds, Sheena Bennet, Asad Bhatty, Gregory Y H Lip, John Camm, Jianhua Wu, Sven Plein, Peter Swoboda, Chris P Gale

**Affiliations:** 1 Leeds Institute of Data Analytics, University of Leeds, Leeds, England, UK; 2 Leeds Institute for Cardiovascular and Metabolic Medicine, University of Leeds, Leeds, UK; 3 Department of Cardiology, Leeds Teaching Hospitals NHS Trust, Leeds, UK; 4 Luliu Hatieganu University of Medicine and Pharmacy, Cluj-Napoca, Romania; 5 Liverpool Heart & Chest Hospital, Liverpool, UK; 6 Department of Clinical Medicine, Aalborg University, Aalborg, Denmark; 7 City St George’s University of London, London, UK; 8 Queen Mary University of London Barts and The London School of Medicine and Dentistry, London, UK

**Keywords:** Atrial Fibrillation, Magnetic Resonance Imaging, Electronic Health Records

## Abstract

**Introduction:**

Atrial fibrosis identified on cardiac magnetic resonance (CMR) imaging has been proposed as a preprocedural imaging biomarker for patient selection for rhythm control interventions in patients with atrial fibrillation (AF). Whether atrial fibrosis is present in patients considered as ‘pre-AF’ is unknown.

**Methods and results:**

We prospectively recruited 12 participants with pre-AF as defined by the Future Innovations in Novel Detection for Atrial Fibrillation (FIND-AF machine learning algorithm, without AF diagnosed during AF screening, and compared them to 25 participants with confirmed AF. All participants underwent CMR using a 3T system with left atrial fibrosis quantification and ADAS-3D left atrial image postprocessing software. Participants with pre-AF had smaller left atrial end-systolic (33.6±9.8 vs 43.0±17.0, p=0.003) and end-diastolic (16.5±8.7 vs 28.2±14.4, p=0.007) volumes, and higher left atrial ejection fraction (59.6±14.6 vs 40.7±17.5, p=0.005) than participants with AF. The extent of atrial fibrosis was not different between those with pre-AF and AF (borderzone (%) 5.2±5.0 vs 2.9±6.9, p=0.772; borderzone fibrosis (cm) 6.2±5.8 vs 6.8±10.7, p=0.927).

**Conclusion:**

CMR identifies atrial fibrosis before manifest AF in patients with pre-AF as defined by a machine learning algorithm.

**WHAT IS ALREADY KNOWN ON THIS TOPIC**

**WHAT THIS STUDY ADDS**

- This study aims to understand cardiac magnetic resonance derived atrial fibrosis and cardiac myopathic changes in patients considered ‘pre atrial fibrillation’ as defined by a machine learning algorithm.

**HOW THIS STUDY MIGHT AFFECT RESEARCH, PRACTICE OR POLICY**

- Atrial fibrosis can play a potential role in early patient identification and introduction of preventative therapies in arresting the manifestation and/or sustenance of atrial fibrillation.

## Introduction

 Myocardial fibrosis is the hallmark of left atrial structural remodelling and has been proposed as a necessary tissue substrate for the maintenance of atrial fibrillation (AF).^1^ Advances in cardiac magnetic resonance (CMR) imaging through delayed gadolinium enhancement have enabled the non-invasive detection of areas likely corresponding to myocardial fibrosis. In the DECAAF study, the presence and increasing magnitude of CMR-identified atrial fibrosis were associated with increased unadjusted and adjusted cumulative incidence of recurrence of AF after catheter ablation.^2^

Data from animal models and human studies show that AF leads to atrial fibrosis,^3 4^ but whether atrial fibrosis can be identified using CMR before the manifestation of overt AF is unknown. Patients with higher predicted risk of AF, as defined by the Future Innovations in Novel Detection for Atrial Fibrillation (FIND-AF) risk score, have a higher incidence of AF compared with lower risk individuals and more frequently have device-detected AF diagnosed on AF screening.^5 6^ Furthermore, patients with higher compared with lower FIND-AF risk have higher indexed left ventricular mass, larger indexed left atrial and ventricular end-diastolic volume and higher native T1 signal and extracellular volume.^7^ Thus, these individuals demonstrate the structural characteristics of a pre-AF phenotype and represent a suitable target population to investigate for the presence of atrial fibrosis before manifest AF.

In this study, we sought to characterise whether CMR-identified atrial fibrosis is present in people with pre-AF, as defined by the FIND-AF score, and compared extent atrial fibrosis to a cohort of patients with clinically manifest AF. We hypothesised that patients with pre-AF would have evidence on CMR of atrial fibrosis.

## Methods

### Study population

### Future Innovations in Novel Detection for Atrial Fibrillation

#### FIND-AFFuture Innovations in Novel Detection for Atrial Fibrillation

FIND-AF is a supervised machine learning multivariable model that predicts the likelihood of developing AF within 6 months for individuals aged ≥30 years based on routinely collected variables including age, sex and comorbidities.^5^ It was developed and internally validated in over 2 million UK secondary care-linked primary care electronic health records.^5^ The included variables are age, sex and the following comorbidities: diabetes mellitus, heart failure, hypertension, stroke or systemic embolism, ischaemic heart disease, chronic obstructive pulmonary disease (COPD), valvular heart disease, chronic kidney disease, rheumatoid arthritis and hyperthyroidism (see supplementary material). In routine retrospective data the area under the receiver operating characteristic curve for a new diagnosis of AF is 0.824.^8^

The FIND-AF longitudinal cohort study is an ongoing multicentre prospective cohort study of patients with pre-AF, those with high FIND-AF risk not found to have AF during ECG screening in the FIND-AF study.^6^ The protocol has been previously published.^8^ Participants were recruited into the FIND-AF study of AF screening between October 2023 and September 2024 with participants aged at least 30 years, without known AF or atrial flutter, and eligible for oral anticoagulation (men with a CHA_2_DS_2_-VASc score ≥2 or women with a CHA_2_DS_2_-VASc score ≥3) (NCT05898165).^5^ Individuals receiving any form of anticoagulation and those on the palliative care register are excluded. To enter the FIND-AF longitudinal cohort study requires participants to be at high risk of AF according to the FIND-AF risk score (FIND-AF scores >0.00834, top 5% of risk in the general population) and not to have received a diagnosis of AF during an AF screening protocol of four ECG recordings per day for 3 weeks using a handheld ECG recorder. Diagnosis of AF was defined as the absence of co-ordinated atrial activity (absence of p waves and irregular R-R intervals) recorded in a single lead ECG of minimum 30 s duration. We also followed up patients with pre-AF at 6 months after initial ECG screening through routinely collected EHRs to establish new AF diagnoses. The FIND-AF study has ethical approval (North West—Greater Manchester South Research Ethics Committee reference 23/NW/0180).

#### The VENTOUX study

We included patients with AF recruited from routine clinical outpatient follow-up who exercised less than 10 hours per week (‘sedentary’) from the VENTricular arrhythmia and cardiac fibrOsis in endUrance eXperienced athletes (VENTOUX) prospective study (recruited between May 2023 and February 2024).^9^ All recruited patients were under routine follow-up in cardiology arrhythmia outpatient service prior to scanning. We did not include any athletes from the VENTOUX study, given that we believed that fibrosis patterns could vary by athlete status.^10^ Ethical approval for the VENTOUX study was granted by the South Yorkshire & Humber NHS Research Ethics Committee and Health Research Authority (21/YH/0231).

### CMR requisition

All scans were performed between April 2023 and May 2024. All CMR scans were undertaken on a 3T system (Siemens Magnetom Prisma, Erlangen, Germany). Participants were advised to avoid caffeine consumption 24 hours prior to their allocated scans. Individuals with estimated Glomerular Filtration Rate (eGFR) <30 mL/min/1.73 cm^2^, with previous experience of claustrophobia or contrast allergy, were excluded. Delineation of epicardial and endocardial borders in short (ventricles) and long (atria) axis was used for the calculation of volumes and mass. All measurements were indexed to body surface area.

The scanning protocol consisted of cine imaging, native and post-contrast T1 mapping using a modified Look Locker sequence, motion corrected automated in line rest perfusion mapping and motion corrected bright blood late gadolinium enhancement. Left ventricle (LV) systolic function was measured through regional and global LV function assessments undertaken via imaging obtained in standard long-axis (two-chamber, three-chamber and four-chamber views) and short axis planes using steady-state free precession pulse sequence; repetition time 3.1–3.3 m, echo time 1.4 ms, flip angle 54°, 8 mm slice thickness, 25 cardiac phases. Likewise, LV volumes (cine imaging) were also acquired through bSSP sequence; repetition and echo time 3.1 ms and 1.4 ms, respectively, 8 mm slice thickness with 2 mm gap, flip angle 52°, matrix 208×140, 25 cardiac phases.

An intravenous bolus of 0.05 mmol/kg gadobutrol (Gadovist, Leverkusen, Germany) was administered 5 mL/s followed by a 20 mL saline flush using an automated injection pump (Medrad MRXperion Injection System, Bayer).

Late gadolinium enhancement was reported if enhancement was identified in two orthogonal planes or both on dark and bright blood late gadolinium enhancement images. T1 and perfusion mapping was analysed using ci42 software (Circle Cardiovascular Imaging, Calgary, Canada). Volumetric parameters such as left and right end systolic and diastolic volumes were recorded after indexing to patient body surface area. This was similar for other parameters such as atrial mass and chamber volumes. All images were reviewed by an imaging cardiologist consultant with the generation of an imaging report for patients with clinically significant cardiac or extracardiac features identified on the research scan. Participants were made aware of these findings on the day of scan by the research team in addition to notification of their primary care team through notification of imaging report.

In addition to CMR imaging protocol, patients underwent left atrial fibrosis quantification using commercially available ADAS-3D left atrial image postprocessing software (GalgoMedical, Barcelona, Spain). Trans-axial LGE images generated underwent quality control assessment for myocardial nulling and artefact detection, with removal of images considered to be of poor quality. Segmented left atrial (LA) images were semiautomatically contoured for LA mid-myocardial walls with manual exclusion of pulmonary vein (PV) ostial points and mitral valve (MV) annulus. A 3D LA model was reconstructed by the software with manual adjustments made using CMR images as reference points to exclude MV annulus and PV. The MV annulus was used as a reference marker to separate the left atrium from the left ventricular cavity, with any enhancement secondary to its structure excluded from final left atrial fibrosis quantification. LA fibrosis was quantified using the image intensity ratio method by comparing LA wall pixel intensity with blood pool signal. Image intensity ratio >1.2 was defined as fibrosis. LA fibrosis percentage was calculated as proportion of enhanced LA considered as fibrosis to total LA area. ‘Borderzone’ was considered the area of LA in transitionary phase between healthy and fibrotic tissue, whereas ‘core’ areas were considered to be purely fibrotic. LA tissue with image intensity ratio >1.1 was considered as ‘borderzone’. The 3D images underwent quality control independently by two experienced users prior to postprocessing, with images deemed poor quality excluded from final analysis.

### Statistical analysis

Subjects were categorised into participants in the Ventoux cohort with diagnosed AF, and participants in the FIND-AF cohort with pre-AF but without diagnosed AF. Continuous variables were expressed as mean (SD), and categorical variables as *n* and percentage. An independent samples T-test was used for comparing proportions between the two groups, which followed a pattern of normal distribution. Alternatively, non-parametric tests such as the Mann-Whitney U test were used to compare variables between two groups that did not follow normal distribution. The χ^2^ test was used for categorical data. Statistical analysis was performed using R Software (Vienna, Austria) and SPSS V.18.0 (Chicago, Illinois, USA). A p value <0.05 was considered to be statistically significant.

### Patient and public involvement

The FIND-AF patient and public involvement group has given their input regarding methods, reporting and dissemination at multiple times throughout the study.

## Results

### Study participant demographics and characteristics

37 participants were enrolled to undergo CMR imaging, 12 with pre-AF and 25 sedentary participants with AF. Among AF participants, 40% (n=10) had persistent AF and 60% (n=15) persistent AF at time of study recruitment and undergoing CMR imaging. Patients with AF were younger and had a lower burden of comorbidities, including hypertension, diabetes mellitus, COPD, chronic kidney disease and ischaemic heart disease (table 1).

**Table 1:** Baseline characteristics

	Total (n=37)	AF (n=25)	Pre-AF (n=12)	P value
Age (years), mean±SD	66.9±8.5	63.9±8.6	73.2±6.0	<0.001
Sex (N, %)				
Female	7 (18.9)	0	7 (58.3)	
Male	30 (81.1)	25 (100)	5 (41.7)	
BMI (kg/m^2^), Mean±SD	28.2±6.24	28.0±7.3	28.6±3.3	0.50
Diabetes mellitus (n, %)	4 (10.8)	1 (4.0)	3 (25.0)	0.30
Hypertension (n, %)	22 (59.5)	11 (44.0)	11 (91.7)	0.05
Valvular heart disease (n, %)	5 (13.5)	3 (12.0)	2 (16.7)	0.80
Heart failure (n, %)	9 (28.1)	8 (32.0)	1 (8.3)	0.30
COPD (n, %)	3 (8.1)	1 (4.0)	2 (16.7)	0.30
Ischaemic heart disease (n, %)	6 (16.7)	2 (8.0)	4 (33.3)	0.10
Chronic kidney disease (n, %)	3 (8.1)	1 (4.0)	2 (16.7)	0.30
Malignancy (n, %)	4 (10.8)	3 (12.0)	1 (8.3)	0.80
Depression (n, %)	2 (5.4)	1 (4.0)	1 (8.3)	0.80

AF, atrial fibrillation; BMI, body mass index; COPD, chronic obstructive pulmonary disease.

### Myocardial structure and function

The full CMR data are given in table 2. Individuals with pre-AF had a higher right ventricular ejection fraction (51.7±7.6 vs 59.6±7.0, p=0.006) but participants with AF had higher left ventricular mass (53.9 ± 7.8vs 50.6 ± 10.4, p=0.33) and larger left atrial volumes (41.7±14.1 vs 33.6±13.4, p=0.11), which was numerically but not statistically higher when indexed to the body surface area. Although the left ventricular end diastolic volume was higher in AF in comparison to the pre-AF cohort (167.0±28.1 vs 149.1±51.0, p=0.04), there was no statistical difference when these volumes were indexed to the body surface area (78.9±12.4 vs 77.5±22.6, p=0.48).

**Table 2:** Cardiac magnetic resonance baseline volumetrics

	Total (n=37)	AF (n=25)	Pre-AF (n=12)	P value
LAVI (mL), mean±SD	39.1±14.2	41.7±14.1	33.6±13.4	0.11
LVEDVI (mL/m^2^), mean±SD	78.5±16.0	78.9±12.4	77.5±22.6	0.48
LVEDVI (mL/m^2^), median (IQR)	75.9 (70-88)	76.1 (61-87)	73.3 (66–79)	0.47
LVESVI (mL/m^2^), mean±SD	35.2±12.5	36.1±10.8	33.1±15.9	0.16
LVESVI (mL/m^2^), median (IQR)	33.0 (27–40)	34.9 (28-41)	27.8 (22–37)	0.16
LVEF (%), mean±SD	55.3±10.7	53.6±11.9	58.9±8.4	0.09
LVMI (g/cm^2^), mean±SD	52.9±9.0	53.9 ± 7.8	50.6 ± 10.4	0.33
RVEF (%), mean±SD	54.3±8.4	51.7±7.6	59.6±7.0	<0.01
RVEDVI (mL/m^2^), mean±SD	80.6±17.1	80.8±17.1	80.0±18.7	0.89
RVEDVI (mL/m^2^), median (IQR)	77.8 (72-86)	78.7 (69-85)	76.7 (73-86)	1.0
RVESVI (mL/m^2^), mean±SD	38.0±10.4	39.4±10.9	34.9±8.0	0.25
RVESVI (mL/m^2^), median (IQR)	37.7 (30-43)	40.5 (31-44)	33.0 (29-41)	0.29
RAVI (mL/m^2^), mean±SD	42.6±18.6	44.2±20.4	39.1±17.9	0.44

AF, atrial fibrillation; BSA, body surface area; LAVI, indexed left atrial volume; LV, left ventricle; LVEDVI, indexed left ventricle end diastolic volume ; LVEF, left ventricular ejection fraction; LVESVI, indexed left ventricle end systolic volume ; LVMI, indexed left ventricular mass; RAVI, indexed right atrial volume ; RVEDVI, indexed right ventricle end diastolic volume; RVEF, right ventricular ejection fraction; RVESVI, indexed right ventricle end systolic volume .

### Left atrial parameters, including atrial fibrosis

Participants with AF had higher left atrial end-systolic (43.0±17.0 vs 33.6±9.8, p=0.003) and end-diastolic (28.2±14.4 vs 16.5±8.7, p=0.007) volumes and lower left atrial ejection fraction (40.7±17.5 vs 59.6±14.6, p=0.002) than participants with pre-AF (table 3).

**Table 3:** Cardiac magnetic resonance features of the left atrium as per ADAS protocol

	Total (n=36)	AF (n=24)	Pre-AF (n=12)	P value
LAESVI (mL), mean±SD	40.4±15.7	43.0±17.0	33.6±9.8	0.003
LAEDVI (mL), mean±SD	24.5±14.0	28.2±14.4	16.5±8.7	0.007
LAEF (%), mean±SD	46.8±18.9	40.7±17.5	59.6±14.6	0.005
BZ+core (cm), mean±SD	6.6±9.2	6.8±10.7	6.2±5.8	0.927
BZ+core (cm), median (IQR)	3.9 (2.5–7.0)	3.8 (2.5–6.4)	4.0 (2.4–6.6)	0.912
BZ+core (%), mean±SD	5.0±6.2	2.9±6.9	5.2±5.0	0.772
BZ+core (%), median (IQR)	3.2 (1.8–5.5)	2.9 (1.8–5.3)	3.8 (2.0–5.5)	0.754

AF, atrial fibrillation; BZ, borderzone fibrosis; LAEDVI, left atrial end systolic volume indexed to body surface area; LAEF, left atrial ejection fraction; LAESVI, left atrial end systolic volume indexed to body surface area (BSA).

**Figure 1:**
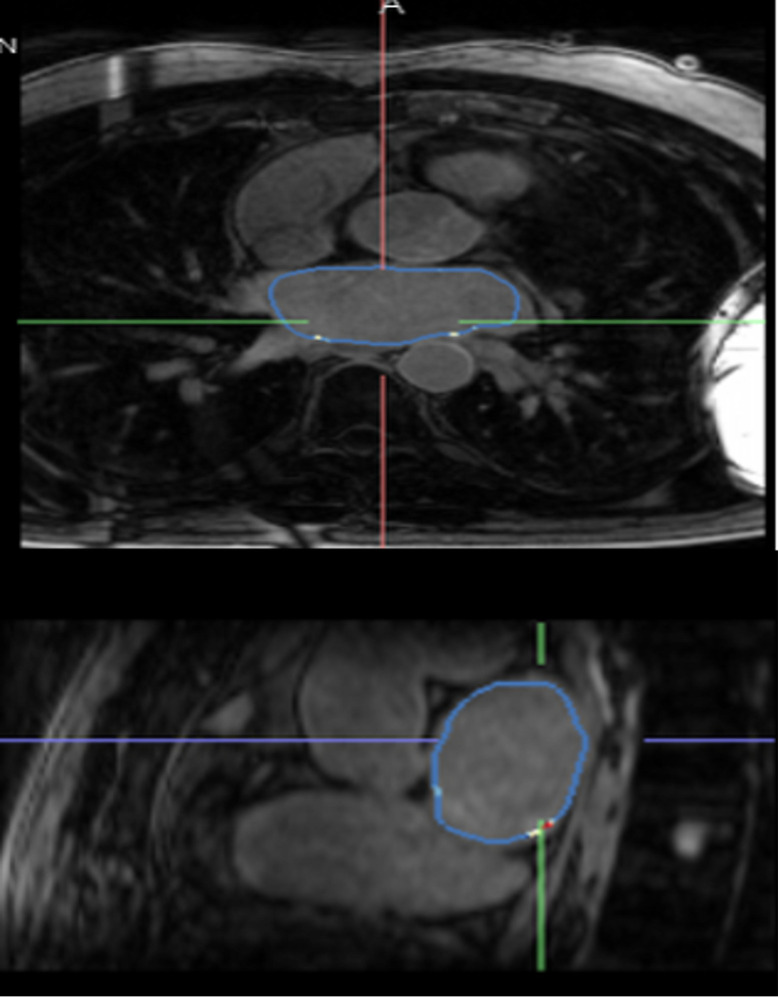
Manual contours drawn on CMR LA cine images to exclude PV and MV. CMR, cardiac magnetic resonance; LA, left aLeft Atrial; MV, mitral valve; PV, pulmonary vein.

**Figure 2:**
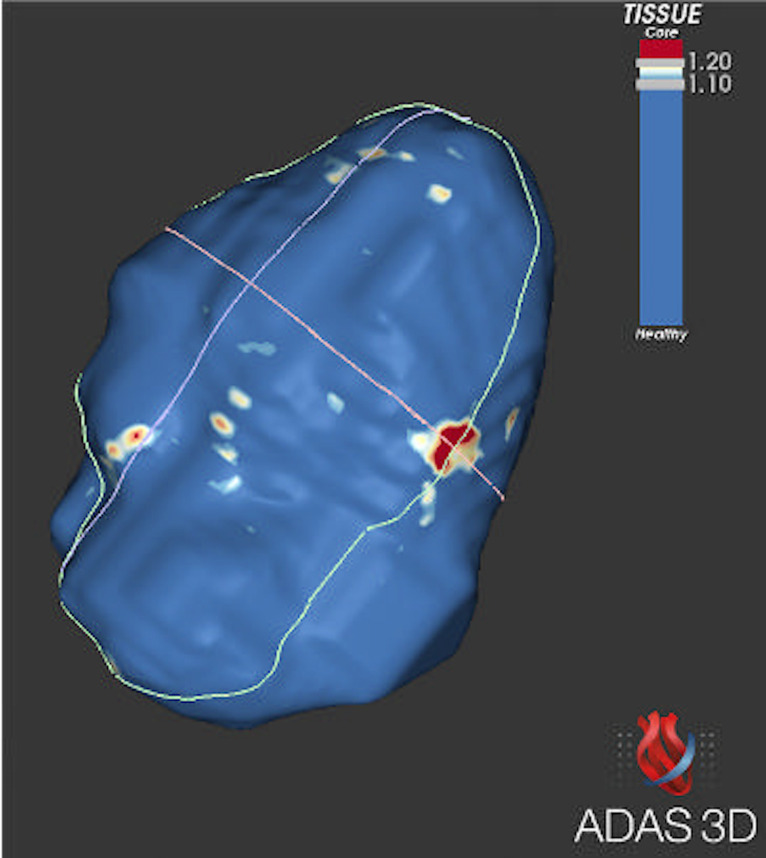
ADAS-3D reconstruction of the left atrium.

**Figure 3:**
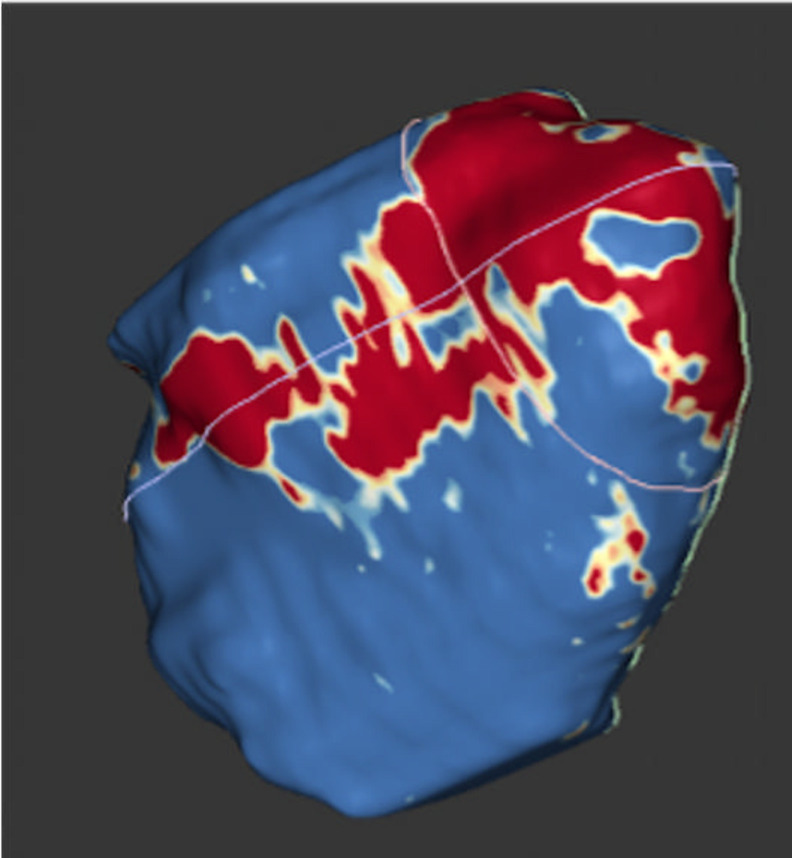
ADAS-3D reconstruction of the left atrium with core and borderzone fibrosis.

The extent of atrial fibrosis was not different between those with AF and those with pre-AF (borderzone fibrosis (cm) 6.8±10.7 vs 6.2±5.8, p=0.927; borderzone (%) 2.9±6.9 vs 5.2±5.0, p=0.772). Manual left atrial contouring (figure 1) and 3D reconstruction through ADAS (figure 2 figure 3).

### Follow-up of patients with pre-AF

Of participants with pre-AF at 6month follow-up after the initial AF screening protocol, there were no patients who had been diagnosed with AF in routine clinical care.

## Discussion

This prospective imaging study demonstrates for the first time in the literature that patients with pre-AF, as defined by a machine learning algorithm, have atrial fibrosis identifiable on CMR. Compared with participants with clinically diagnosed AF, participants with pre-AF demonstrated smaller atrial volumes and higher atrial ejection fraction.

Atrial fibrosis has been proposed as a preprocedural imaging biomarker for patient selection for rhythm control interventions in patients with AF. In the prospective, observational DECAAF study of 272 patients with paroxysmal or permanent AF undergoing first catheter ablation, a greater degree of atrial fibrosis detected on delayed enhancement cardiac magnetic resonance was associated with arrhythmia recurrence.^2^ Furthermore, in a retrospective observation analysis of 47 patients undergoing a maze operation during MV surgery, those who did not have a successful restoration of sinus rhythm had a higher degree of left atrial fibrosis compared with those who did have successful restoration of sinus rhythm after adjustment for confounders.^11^

Atrial fibrosis is thought to be essential to the maintenance of atrial arrhythmias and to increased AF burden, as demonstrated in experimental animal models.^12 13^ In the pathological conditions associated with the development of fibrosis, fibroblasts may proliferate and differentiate into myofibroblasts that may directly slow conduction.^14^ This makes the architecture of the fibrotic tissue more heterogeneous, affecting intercellular conduction and increasing anisotropy,^15 16^ leading to slower electrical conduction and overall creation of an arrhythmogenic substrate. Computational modelling has suggested that fibrosis-induced disorganisation of electrotonic coupling may not just maintain arrhythmias but also lead to increased automaticity and atrial ectopy, thus potentiating arrhythmia triggering mechanisms.^17 18^

The hypothesis-generating data suggest that atrial fibrosis may precede manifestation of clinical AF in patients with comorbidities predisposing to AF. There was also evidence of more advanced atrial remodelling in patients with AF compared with pre-AF, with larger left atrial volumes and lower atrial ejection fraction. This could be interpreted that atrial fibrosis may be an intermediate step in the manifestation of atrial myopathy and then AF. However, the participants with clinically diagnosed AF were all men, whereas more than half of the pre-AF cohort were women, which may affect interpretation of left atrial size.^19^ Furthermore, whether atrial fibrosis is present only in patients with higher predicted risk of AF or more generally in older patients is unknown. In addition, none of the 12 participants with pre-AF were diagnosed with AF over the following 6 months in routine care. A future study could assess atrial fibrosis and conduct longer term continuous ECG monitoring in age-matched and sex-matched groups of low AF risk, high AF risk and clinically manifest AF to provide more definitive evidence for the role of atrial fibrosis before clinical manifestation of AF. As the global burden of AF and its sequelae rises,^20 21^ the early detection of AF, or altering AF genesis, is a research priority,^22^ and early substrate identification in AF could guide targeted pathways for AF detection and prevention or act as a treatment target in randomised clinical trials.

### Limitations

We recognise limitations in our current study. First, this was a proof-of-concept study to determine whether atrial fibrosis was present in patients at pre-AF in sinus rhythm. The small sample size reduces the power to detect relevant differences for comparison of atrial fibrosis quantification and chamber volumetrics between participants with pre-AF and with AF. A larger study is required to determine the robustness of these findings. Second, quantification of atrial fibrosis can be affected by spatial resolution with the challenges in imaging the thin atrial wall and motion artefact during the cardiac cycle and motion artefacts due to breathing can affect the quality of images acquired. However, we used published methods and software to quantify atrial fibrosis.^23^ Third, we used IIR1.2 and 3SD (above the mean blood pool signal intensity as a reference) for atrial fibrosis quantification.^1 24^ Despite multiple thresholds explained in literature, there is still no consensus, standardisation or external validation in these quantification methods. Fourth, patients with pre-AF do not have routinely detected AF and were not found to have AF during intermittent ECG monitoring four times a day for 30 s over 3 weeks, a standardised AF screening protocol.^25^ However, it is possible that some of these participants may have short runs of subclinical AF detected if they underwent continuous monitoring. Fifth, observers reporting the CMR scans were not blinded to the group allocation of the participant, as participants were recruited asynchronously between the two cohorts. This has the potential to introduce bias. Sixth, the patients with AF recruited from routine care were all male, with a median age of 63 years, and thus are not representative of the general AF population.^20^ Furthermore, they were younger and had a higher burden of comorbidities than the participants with pre-AF. Advancing age may contribute to atrial fibrosis,^26^ and there may be sex-based differences in atrial characteristics and fibrosis patterns.^27^ Thus, the findings in this study need to be tested in age-matched and sex-matched participants with AF and pre-AF.

## Conclusion

In this prospective study, we demonstrate the presence of atrial fibrosis before the presence of AF in patients with pre-AF as defined by a machine learning algorithm. CMR-derived atrial fibrosis may have utility in identifying patients with atrial myopathic changes before the manifestation of AF.

## Supplementary material

10.1136/openhrt-2025-003747 online supplemental file 1

## Data Availability

Data are available upon reasonable request.
