# Supplementary material for: Cardiac magnetic resonance imaging-derived atrial fibrosis in patients with pre-atrial fibrillation
Source: Open Heart. 2025 Nov 27;12(2):e003747. doi: 10.1136/openhrt-2025-003747 (PMC12666032; doi:10.1136/openhrt-2025-003747)
Supplement: online supplemental file 1 [file openhrt-12-2-s001.docx]

**Supplementary appendix**

Atrial fibrosis in patients with pre-atrial fibrillation and manifest atrial fibrillation

Ali Wahab, Ramesh Nadarajah, Raluca Tomoia, Wasim Javed, Catherine Reynolds, Sheena Bennett, Asad Bhatty, Gregory Y H Lip, A John Camm, Jianhua Wu, Sven Plein, Peter Swoboda, Chris P Gale

**Future Innovations in Novel Detection of Atrial Fibrillation (FIND-AF)**

| **Components of FIND AF score** |
| --- |
| Age |
| Sex |
| Ethnicity |
| Hypertension |
| DM |
| COPD |
| Valvular Heart Disease |
| Vascular (IHD/ previous MI/PVD) |
| Stroke |
| Heart Failure ( HfPEF / HFrEF) |
| Hyperthyroidism |

COPD- Chronic Obstructive Pulmonary disease, DM- Diabetes Mellitus, HfPEF- Heart Failure with Preserved Ejection Fraction, HFrEF- Heart Failure with Reduced Ejection Fraction, IHD- Ischaemic Heart Disease, MI- Myocardial Infarction, PVD- Peripheral Vascular Disease

Scores >/= 0.00534 considered ‘high risk’
